# A bilateral cervical accessory biceps muscle: anatomical description and clinical implications

**DOI:** 10.1007/s00276-026-04038-y

**Published:** 2026-10-16

**Authors:** Maria Luísa Rodrigues Oliveira Roque, Lorenzo Ribeiro Dias Vieira, Alice Viçosi da Silva, Josemberg da Silva Baptista

**Affiliations:** Laboratory of Applied Morphology (LEMA), Department of Morphology, Universidade Federal do Espírito Santo, Av. Marechal Campos, 1468, Campus de Maruípe Vitória, Vitória, Espírito Santo 29040-090 Brazil

**Keywords:** Anatomic variation, Back muscles, Cadaver, Dissection, Neck muscles

## Abstract

**Purpose:**

Accessory muscles between the levator scapulae and serratus posterior superior are rarely reported and may be interpreted as variants of either adjacent muscle. This study describes bilateral accessory muscles in the posterior cervical and upper thoracic region and discusses their anatomical classification and clinical relevance.

**Methods:**

A routine anatomical dissection was performed in a formalin-fixed adult male cadaver. The posterior cervical and upper thoracic regions were dissected to define the topography, attachments, morphology, distal interface, and innervation of the accessory muscles. Morphometric measurements were obtained bilaterally using a caliper.

**Results:**

Bilateral elongated muscular structures were identified between the levator scapulae and serratus posterior superior muscles. Each muscle had an individual proximal attachment at the transverse process of the second cervical vertebra, followed an inferomedial course approximately parallel to the splenius capitis, and remained distinct from the main belly of the levator scapulae. Distally, each muscle was separated from the serratus posterior superior by a bursal interface, without direct muscular or aponeurotic continuity or independent scapular insertion. Both muscles were bicipital and fusiform and were innervated by posterior rami that also supplied the splenius capitis.

**Conclusion:**

These findings support the interpretation of the structures as paired individualized accessory muscles rather than accessory slips of adjacent muscles, expanding the known spectrum of posterior cervical and upper thoracic anatomical variations.

## Introduction

Considerable variation has been reported in the origins, course, number of slips, and distal attachments of the levator scapulae (LS). Accessory attachments may reach muscles such as the rhomboids, trapezius, serratus anterior, serratus posterior superior (SPS), thoracolumbar fascia, and clavicle, upper ribs, and thoracic spinous processes [1, 10]. These findings demonstrate that accessory slips of the LS may establish continuity with structures located in different muscular and myofascial planes of the posterior cervical and upper thoracic regions.

Connections between the LS and SPS have received different anatomical interpretations. Loukas et al. and Kim and Lee described accessory heads arising in continuity with the LS and inserting, through broad aponeurotic bands, into the SPS and additional structures [5, 8]. In contrast, Lashley and Granite described an anomalous muscle inserting onto the SPS and interpreted it as a possible accessory SPS rather than a variant of the LS [6].

However, the anatomical classification of accessory structures located between the LS and SPS remains unclear when they are not continuous with either adjacent muscle and show features suggesting an individualized muscular structure.

The present report describes bilateral accessory muscles between the LS and SPS muscles in an adult male cadaver. Their bilateral occurrence, fusiform morphology, posterior ramus innervation, proximal attachment at C2, and distal bursal interface with the SPS provide an opportunity to discuss their anatomical classification and possible placement within the superficial post-transversarium stratum.

## Materials and methods

A routine dissection of the posterior cervical and upper thoracic regions of a formalin-fixedadult male cadaver, aged approximately 50–60 years, was performed at the Laboratory of Applied Morphology, Universidade Federal do Espírito Santo, Vitória, Espírito Santo, Brazil. The specimen was obtained through an institutional body donation program in accordance with Brazilian Law No. 8.501/1992.

During the dissection of the serratus posterior superior, an unusual muscular structure was identified bilaterally. Each structure was carefully dissected along its entire visible course to define its proximal attachment, distal relationship with the SPS, and general morphology. The adjacent LS, SPS, and splenius capitis muscles were preserved to allow topographic comparison. Small nerve branches entering each accessory muscle were identified under direct visualization, preserved, and followed proximally as far as possible to determine their relationship with the posterior rami supplying the adjacent splenius capitis muscle.

Morphometric measurements were obtained using a caliper (King Tool, China). The maximum length and width of each accessory muscle were recorded bilaterally. Digital photographs were obtained in posterior and close-up views to document the anatomical relationships, muscular architecture, and distal interface.

## Case report

**Fig. 1:**
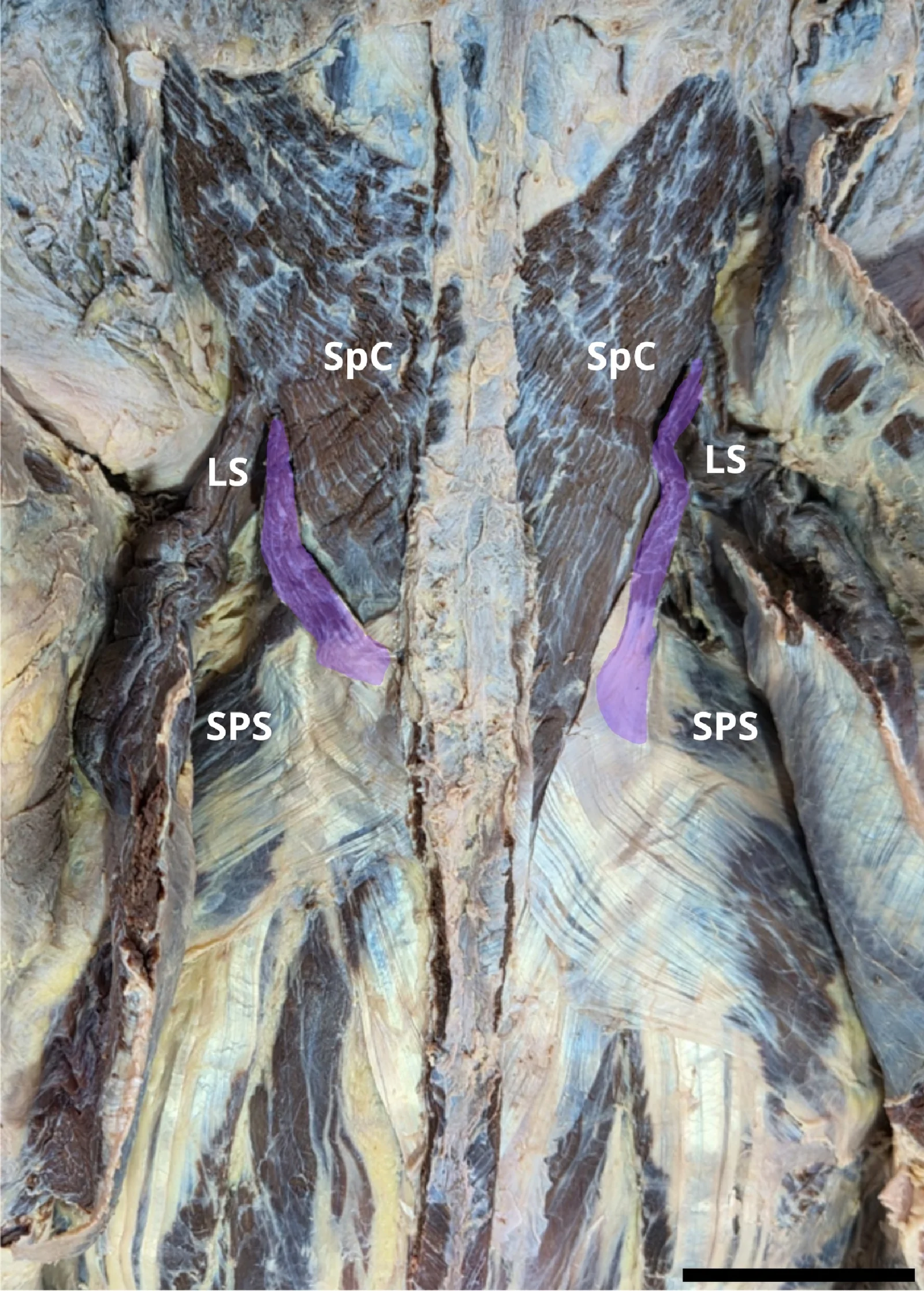
Overview of the bilateral accessory muscles in the posterior cervical and upper thoracic region. Posterior view showing the bilateral accessory muscles located between the levator scapulae and serratus posterior superior muscles. The accessory muscles are highlighted in purple. LS, levator scapulae; SpC, splenius capitis; SPS, serratus posterior superior. Scale bar = 5 cm

Bilateral elongated muscular structures were observed in the superior dorsal region, occupying the interval between the LS and SPS muscles (Fig. 1).

On each antimere, the accessory muscle had a proximal attachment at the transverse process of C2, anterior to the LS muscle. From this attachment, it followed an inferomedial course toward the SPS muscle, running in parallel to the adjacent fibers of the splenius capitis. The main belly of the LS remained clearly distinguishable and continued toward its usual scapular insertion.

**Fig. 2:**
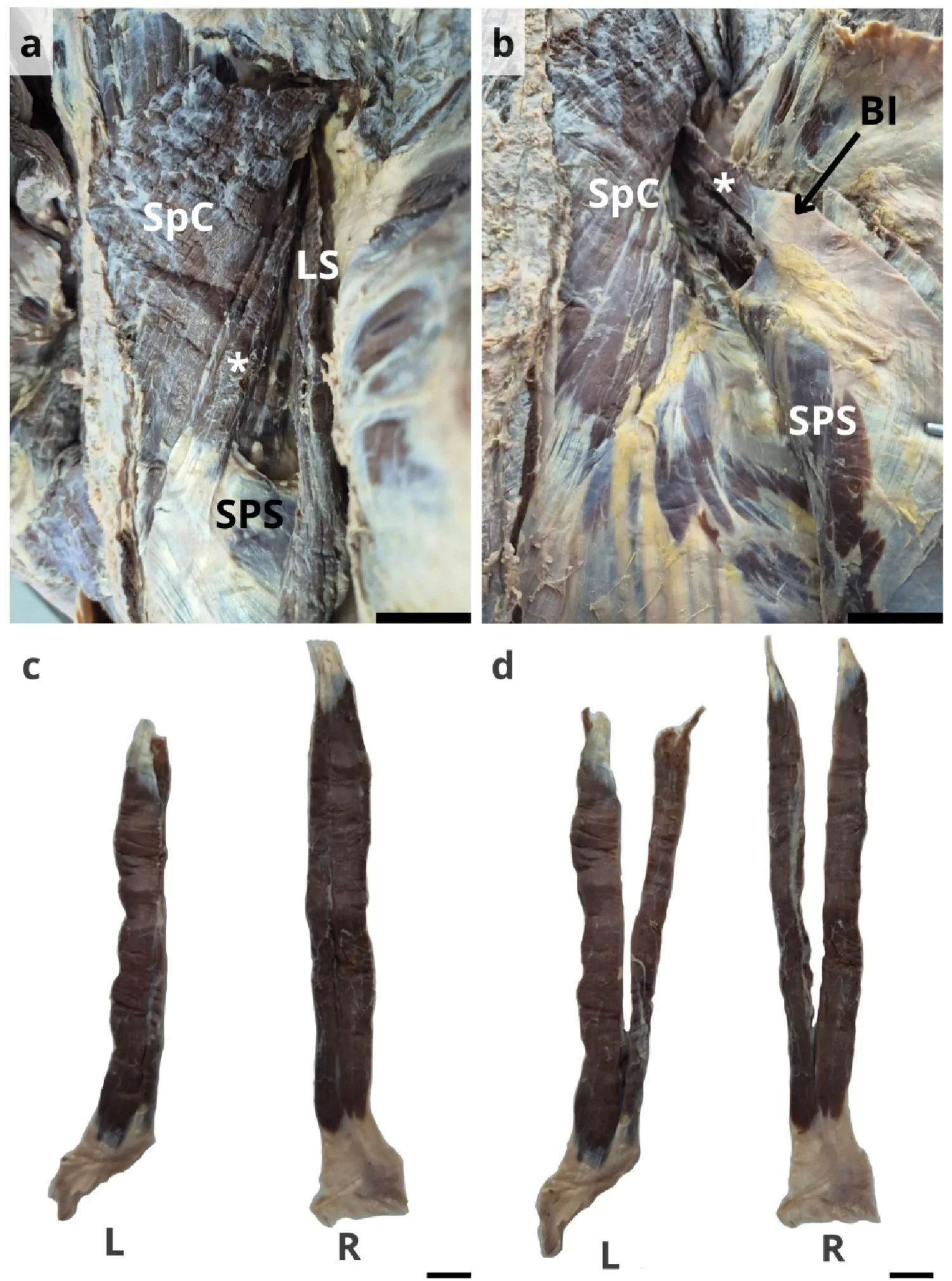
Detailed anatomical views of the bilateral accessory muscles. **a** Right-sided close-up view of the accessory muscle in situ, showing its relationship with the splenius capitis, levator scapulae, and serratus posterior superior muscles. **b** Right-sided detail of the distal region, showing the bursal interface between the accessory muscle and the serratus posterior superior. **c** Isolated right and left accessory muscles after dissection, demonstrating their fusiform morphology and relative size. **d** Isolated accessory muscles showing their bicipital configuration. Asterisks indicate the accessory muscles in panels **a** and **b**. BI, bursal interface; LS, levator scapulae; L, left; R, right; SpC, splenius capitis; SPS, serratus posterior superior. Scale bars = 3 cm in panels a and b, and 1 cm in panels c and d

Inferiorly, a macroscopically identifiable bursal interface was observed between the accessory muscle and the serratus posterior superior muscle, indicating a close anatomical relationship without direct muscular or aponeurotic continuity (Fig. 2a, b). The term bicipital is used here descriptively to indicate that each accessory muscle showed two distinguishable muscular slips forming an elongated fusiform structure after isolation (Fig. 2c, d). No independent direct insertion onto the scapula was identified. Small nerve branches entering the accessory muscles were preserved and traced proximally as far as possible; these branches were continuous with posterior ramus branches that also supplied the adjacent splenius capitis muscle. The right-sided muscle measured 109.86 mm in length and 14.59 mm in maximum width, whereas the left-sided muscle measured 100.40 mm in length and 11.11 mm in maximum width.

## Discussion

The main finding of the present case was the presence of bilateral accessory muscles between the LS and SPS muscles. Each structure had an individual proximal attachment at C2, anterior to the LS, followed an inferomedial course, and was separated distally from the SPS by a bursal interface. Classical and encyclopedic descriptions of levator scapulae variation have long recognized that accessory slips may extend beyond the usual scapular insertion and establish relationships with adjacent cervicoscapular structures. Macalister described slips of the levator anguli scapulae inserting with the serratus posticus superior, as well as slips reaching the splenius capitis and scalene muscles [9]. Le Double also referred to fascicles of the angulaire de l’omoplate following variable courses toward muscles, aponeuroses, or bones of the neck [7]. Bergman’s account further summarizes dorsal and ventral deviant fasciculi of the levator scapulae, including fasciculi inserting into the serratus posterior superior, rhomboids, spinous processes, and subscapularis [2]. These classical observations support interpreting the present finding within the broader spectrum of cervicoscapular muscular variation. However, unlike the classical descriptions of simple accessory slips, the present structures were bilateral, individualized, bicipital and fusiform, and showed posterior ramus innervation and a distinct bursal interface with the SPS. Therefore, the descriptive term “biceps cervicodorsalis” (BCD) is used here only to discuss the present finding, rather than to establish a formal new anatomical entity.

The cases reported by Loukas et al. and Kim and Lee provide the closest anatomical comparisons with the present findings [5, 8]. In both reports, the LS was divided into a typical lateral head and an accessory medial head that followed an inferomedial course, approximately parallel to the splenius muscles, and widened into a thick aponeurotic band. These bands attached to the SPS and additional structures, including the nuchal ligament, cervical spinous processes, and rhomboid tendons. Both structures were interpreted as accessory heads of the LS because of their continuity, shared cervical origin, and innervation by a branch of the dorsal scapular nerve.

Despite some similarities, the present case is bilateral – no cadaver reports could be found in the literature to compare these data; the BCD displayed a discrete, bicipital, and fusiform morphology; each BCD had an individual attachment at C2 and was separated distally from the SPS by a bursal interface, without additional attachments to the nuchal ligament, cervical spinous processes, or rhomboid tendons.

Only through magnetic resonance imaging Au et al. report caudal accessory attachments of the LS, including bilateral attachments and two cases with bilateral attachments to the SPS [1]. These findings indicate that bilateral accessory relationships between the LS and SPS region can occur. Nevertheless, the present case differs by showing individualized bicipital muscles, posterior ramus innervation, and a bursal interface with the SPS, features not sufficient to classify them simply as accessory LS slips.

Conversely, the distal relationship with the SPS and the absence of an independent scapular insertion could suggest an accessory component of the SPS. Lashley and Granite described a muscle originating from C1 and inserting onto the proximal portion of the SPS, without continuity with the scapula, and classified it as a possible accessory SPS [6]. However, in the present case, the cervical course, posterior ramus innervation, and bursal interface separating each BCD from the SPS support its individuality as an adjacent structure rather than direct fusion or incorporation into that muscle.

Therefore, the presented muscles do not fully conform to either conventional interpretation as simple accessory slips of the LS or as direct accessory components of the SPS. Within the framework of the spinal muscle stratification system described by de Araújo Baptista et al., the present structures are anatomically compatible with the superficial post-transversarium (SPT) stratum [3]. This interpretation is supported by their posterior cervical and upper thoracic topography, proximal axial attachment at C2, inferomedialcourse approximately parallel to the splenius capitis, and innervation by posterior rami that also supplied the adjacent splenius capitis. In addition, the absence of an independent scapular insertion and the presence of a distal bursal interface with the SPS argue against interpreting these structures simply as part of the LS or as a direct fusion with the SPS. Thus, their placement within the SPT stratum is proposed as an anatomical interpretation based on topography, fiber orientation, axial fixation, and innervation, rather than as definitive evidence of a new formal muscular entity. From a developmental perspective, myotube guidance and attachment-site selection are recognized mechanisms involved in the formation of muscle patterns and their connections to specific targets [11]. Disturbances in these processes may therefore provide a possible explanation for the unusual course and attachments observed in the present case. However, this interpretation remains hypothetical in the absence of histological or embryological evidence.

The main limitations of this report are the absence of histological confirmation of the bursal nature of this interface and the restriction of the finding to a single cadaveric case. Therefore, the interpretation proposed here should be regarded as descriptive and anatomical, rather than as definitive evidence of a distinct formal muscular entity.

Accessory muscular bands in the posterior cervical and upper thoracic regions may remain asymptomatic and unnoticed [12]. However, their presence may be relevant during posterior cervical surgery and scapular procedures [4]. Accessory cervical muscles may additionally be mistaken for soft-tissue masses or other pathological structures on imaging examinations [12]. Awareness of their possible bilateral occurrence may help radiologists distinguish symmetrical muscular variants from pathological lesions [1].

## Conclusion

The location, individual proximal attachment, fusiform aspect, posterior ramus innervation, and fiber orientation favor their interpretation as paired accessory muscles rather than as accessory slips of either adjacent muscle. These findings expand the known morphological spectrum of accessory musculature in the posterior cervical and upper thoracic regions.

## Data Availability

No datasets were generated or analysed during the current study.
